# Asymmetric gene introgression in two closely related *Orchis* species: evidence from morphometric and genetic analyses

**DOI:** 10.1186/1471-2148-12-178

**Published:** 2012-09-12

**Authors:** Hans Jacquemyn, Rein Brys, Olivier Honnay, Isabel Roldán-Ruiz

**Affiliations:** 1 Division of Plant Ecology and Systematics, Biology Department, University of Leuven, Kasteelpark Arenberg 31, Heverlee, B-3001, Belgium; 2 Research Institute for Nature and Forest, Brussels, 1070, Belgium; 3 Plant Sciences Unit, Institute for Agricultural and Fisheries Research ILVO, Caritasstraat 21, Melle, B-9090, Belgium

**Keywords:** Admixture, Hybrid swarm, *Orchis*, Orchidaceae, Reproductive isolation

## Abstract

**Background:**

In food-deceptive orchids of the genera *Anacamptis*, *Neotinea* and *Orchis* floral isolation has been shown to be weak, whereas late-acting reproductive barriers are mostly strong, often restricting hybridization to the F1 generation. Only in a few species hybridization extends beyond the F1 generation, giving rise to hybrid swarms. However, little is known about the abundance of later-generation hybrids and what factors drive their occurrence in hybrid populations. In this study, molecular analyses were combined with detailed morphological measurements in a hybrid population of two closely related *Orchis* species (*Orchis militaris* and *O. purpurea*) to investigate the hypothesis that the abundance of later-generation hybrids is driven by changes in floral characters after hybridization that exert selective pressures that in turn affect hybridization.

**Results:**

Both the molecular and morphological data point to extensive genetic and morphological homogenization and asymmetric introgression. Estimating genomic clines from the multi-locus genotype data and testing for deviation from neutrality revealed that 30 out of 113 (27%) AFLP markers significantly deviated from neutral expectations. Plants with large floral displays or plant with flowers that resembled more *O. purpurea* had higher female fitness than plants with small floral displays or plants with flowers resembling more *O. militaris*, suggesting that directional selection may have contributed to the observed patterns of introgression.

**Conclusions:**

These results indicate that in closely related orchid species hybridization and gene introgression may be partly driven by selection for floral traits of one of the parental types. However, because some pure individuals were still present in the studied population, the parental species appeared to be sufficiently isolated to survive the challenge of sympatry.

## Background

The maintenance of species integrity between sexually compatible sympatric populations largely depends on several reproductive barriers that together determine the reproductive isolation acting between species [1]. Isolating barriers act sequentially and are conveniently classified in pre-mating (spatial segregation, phenology and pollinators) and post-mating barriers (fruit abortion, seed inviability, hybrid inviability and hybrid sterility) [2,3]. Because of the sequential action of these isolating mechanisms, it is generally assumed that pre-mating isolation barriers are more important to reproductive isolation than post-mating barriers, although conclusive evidence for this is still largely lacking [1].

Comparative analyses of pre- and post-mating barriers in a wide range of species of the food-deceptive orchid genera *AnacamptisNeotinea* and *Orchis* have revealed relatively weak pre-mating isolation barriers [4]. This is because most food-deceptive orchid species are pollinated by generalist pollinators – most often bees and bumblebees – and most orchid species show considerable overlap in their pollinator community [4-6]. On the other hand, early-acting post-mating barriers appeared to be much stronger [4], suggesting that these barriers played a significant role during speciation and still play an important role in the maintenance of the species’ identity. Late-acting post-mating barriers (hybrid inviability and sterility) were shown to contribute further to reproductive isolation in food-deceptive species [7]. In 56% of potentially hybridizing species, hybrid inviability has been reported and in most crosses involving hybrid individuals reduced fertility was found, suggesting that intrinsic post-mating isolation strongly contributes to the maintenance of reproductive boundaries among these species [7].

Notwithstanding hybridization has been frequently observed in the genera *AnacamptisNeotinea* and *Orchis*[8], it can be expected that due to the cumulative effects of post-mating barriers [7] hybridization in natural populations should in most cases be restricted to the F_1_ generation. As a result, introgression should be rare and species boundaries well-conserved. Most studies investigating the extent of hybridization using molecular markers have indeed shown little crossbreeding beyond the F_1_ generation [9]. For example, in two species of the genus *Anacamptis* (*Anacamptis morio* and *A. papilionacea*), molecular analyses based on nuclear ITS and AFLP markers showed that all examined hybrids were F_1_ hybrids [10]. These results were corroborated by hand-pollination experiments, which showed that hybrids produced no viable progeny, indicating that late-acting post-mating barriers (hybrid sterility) prevented gene introgression [10]. Similarly, most hybrids between *Orchis mascula* and *O. pauciflora* and between *O. italica* and *O. anthropophora* belonged to the F_1_ generation and very few putative backcross individuals were present [11,12]. Hand-pollination experiments also confirmed the low viability of progeny originating from backcross pollinations [11,12]. On the other hand, recent analyses investigating hybridization in the sister species *Anacamptis morio* and *A. longicornu* have shown that reproductive barriers were insufficient to prevent genomic admixture [13], suggesting that in closely related species pre- and post-mating barriers were insufficient to maintain species boundaries. Similarly, analyses of pre- and early acting post-mating barriers acting between the closely related *Orchis militaris* and *O. purpurea* have shown that reproductive isolation, including pollinator sharing, fruit abortion, seed abortion and seed mortality, was low compared to the average reproductive isolation reported for the genus [14] and smaller for crosses between *O. militaris*^♀^ and *O. purpurea*^♂^ (*RI* = 0.42) than for crosses between *O. purpurea*^♀^ and *O. militaris*^♂^ (*RI* = 0.64) [15]. Given that both species display considerable overlap in flowering time and often grow in the same area, the observed degree of reproductive isolation may be insufficient to prevent interspecies mating and thus genomic admixture. However, at present little is known about the frequency of later-generation hybrids and what factors drive their occurrence in hybrid populations.

Previous analyses using AFLP markers have provided evidence of asymmetric introgression in a sympatric population of *O. militaris* and *O. purpurea* in Belgium [16], but the mechanism causing this pattern of introgression remains unclear. One hypothesis may be that interactions with animal pollinators are the primary source for the observed trend towards more *O. purpurea*-like plants. In this case, it can be predicted that female fitness of *O. purpurea*-like plants is higher than that of *O. militaris*-like plants. To test this hypothesis, we combined molecular analyses with morphological measurements to estimate the extent of hybridization and introgression in the same sympatric population of *Orchis purpurea* and *O. militaris* in Belgium. More in particular, data from allopatric populations were included in the molecular analyses to investigate in more detail the extent of introgression and genome-wide admixture. We also assessed genomic clines and tested for deviations from neutral expectations. Finally, the morphometric data were related to fitness data (fruit set and seed viability) to assess selection through female function on floral traits in the hybrid population.

## Results

### Genetic diversity and differentiation

**Figure 1:**
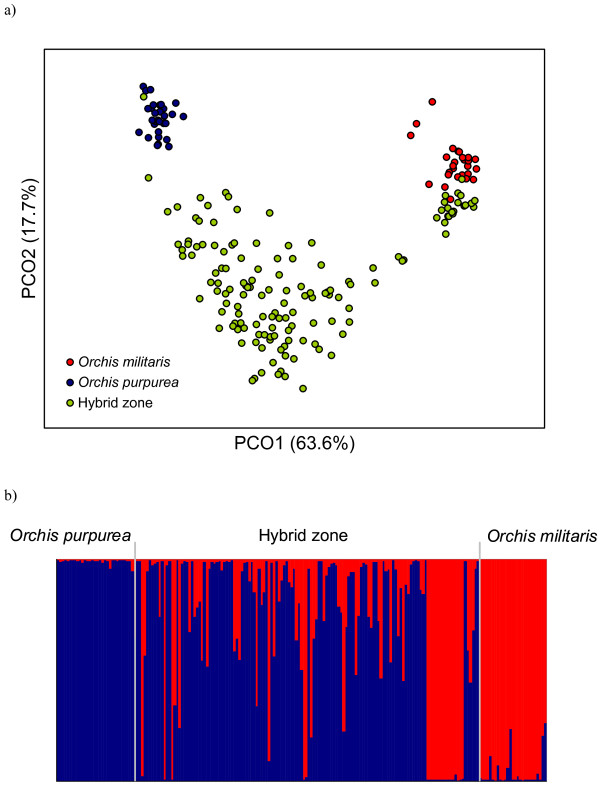
**Clustering analysis of AFLP data for a pure *Orchis militaris *population, pure *O. purpurea *population and a hybrid population.** (**a**) Principal coordinate (PCO) plot based on individual genetic distance calculated with 113 AFLP markers. The first two axes explain 63.6% and 17.7% of the variation, respectively. (**b**) Clustering analysis using structure. Individuals are represented by columns, with colours representing the proportion of their genome assigned to the *K* = 2 inferred clusters in the model-based admixture analysis.

The three primer combinations generated a total of 113 polymorphic bands, of which 99 were polymorphic in the admixed population. Each individual displayed a unique banding pattern. Genetic diversity was consistently higher in the hybrid zone than in the pure populations (Table 1). Genetic differentiation was high (overall *F*_ST_ = 0.41, *P* < 0.001). Pairwise *F*_ST_ values were high between the pure *O. militaris* and *O. purpurea* populations (*F*_ST_ = 0.62), but substantially lower between the hybrid population and the pure *O. militaris* (*F*_ST_ = 0.25) and *O. purpurea* (*F*_ST_ = 0.30) population. The PCoA identified three groups: i) pure *O. purpurea* individuals, ii) pure *O. militaris* individuals, and iii) a large cluster of putative hybrids (Figure 1a). However, not all individuals sampled in the hybrid population appeared to be hybrids, and some individuals clearly clustered within the pure *O. militaris* group. Very few individuals of the hybrid zone clustered within the pure *O. purpurea* group (Figure 1a). Bayesian admixture analyses using Structure yielded similar results. The likelihood (Ln P(D)) increased greatly between *K* = 1 and *K* = 2, but less so after *K* = 2, which, together with the fact that Δ*K* reached its maximum at *K* = 2 (see Additional File 1) suggests the existence of two clusters. Population clustering at successively higher *K* values demonstrated a consistent pattern indicating that individuals from pure *O. militaris* and *O. purpurea* populations formed largely independent clusters with hybrids exhibiting an admixed genotype (see Additional File 1). Pure populations of *O. purpurea* and *O. militaris* were almost entirely composed of purebreds (mean proportion of membership in the two alternative clusters was 0.99 for the *O. purpurea* population and 0.96 for the *O. militaris* population; Figure 1b). The genotypes of both parental taxa contributed to the genotypes in the hybrid population, but despite of the detection of a substantial number of pure *O. militaris* plants, individuals of the admixed population showed a larger contribution of the *O. purpurea* genome (0.72) (Figure 1b), which is in line with previous analyses [16]. 

**Table 1:** **Genetic diversity (*P*: percentage polymorphic loci,* H ***_**j**_**: expected heterozygosity) in pure *O. purpurea *and *O. militaris *populations and in the hybrid zone**

	**Population**		
	***Orchis militaris*(*n* = 27)**	**Hybrid zone(*n* = 140)**	***Orchis purpurea*(*n* = 30)**
*P*	61.1	78.8	59.3
*H*_j_	0.21	0.31	0.15

### Genome-wide admixture and hybrid indices

**Figure 2:**
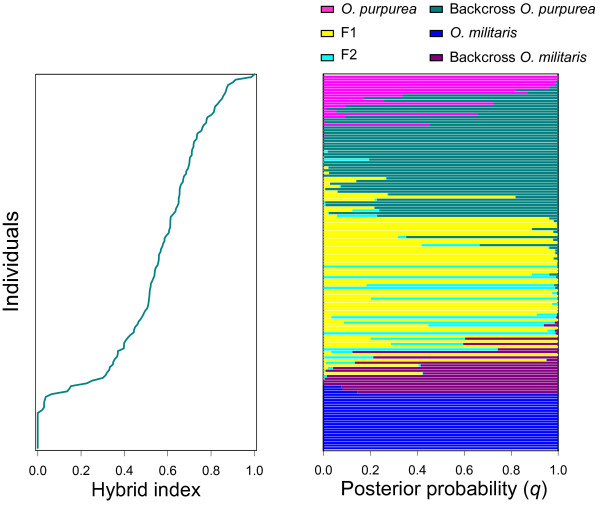
**Hybrid indices calculated for 140 individuals sampled in a hybrid population consisting of two closely related food-deceptive orchid species* Orchis militaris *and* O. purpurea *.** (**a**) This plot gives the fraction the genome inherited from *Orchis purpurea* for 140 individuals. A value of 0 indicates pure *O. militaris* and 1 indicates pure *O. purpurea*. Individuals are ordered by increasing hybrid indices. (**b**) Bayesian inference of genotype class estimated with NEWHYBRIDS. The genotype classes are represented by colors, and individuals are represented as rows. Within each row (individual) the extent of the component colors indicates the posterior probability of an individual with respect to each genotype class.

Hybrid indices for plants of the hybrid populations varied between 0 (pure *O. militaris*) and 1 (pure *O. purpurea*) (Figure 2a). However, the results indicated that the frequency of hybrid indices was significantly skewed to the left, implying that a substantial amount of pure *O. militaris* individuals was present in the population (21 plants carried more than 99% of *O. militaris* in their genome), whereas only one plant had > 99% of *O. purpurea* in its genome (Figure 2a). On the other hand, more than half of all individuals (61.1%) had a hybrid index larger than 0.5, indicating that most individuals had a larger fraction of *Orchis purpurea* in their genome. There was also an abrupt change in hybrid indices between 0.1 and 0.3, after which changes in hybrid indices leveled off (Figure 2a).

Analyses using NEWHYBRIDS and a threshold *q*-value of 0.9 showed that 106 out of 140 (75.7%) individuals sampled in the hybrid population were unequivocally assigned to pure *O. militaris*, pure *O. purpurea* or the four predefined hybrid classes (F1, F2 and backcrosses) (Figure 2b). In total, 36 F1, 31 backcrosses with *O. purpurea*, eight backcrosses with *O. militaris* and five F2 genotypes were identified (Figure 2b). Twenty one pure *O. militaris* plants were found, whereas only five pure *O. purpurea* individuals were observed.

**Figure 3:**
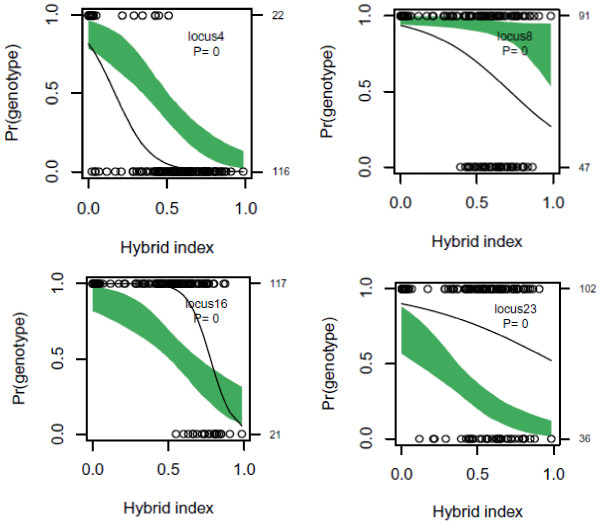
Genetic clines of four example loci that significantly deviated from neutral expectations.

Estimates of genomic clines for each marker locus across the hybrid population revealed that, after correcting for multiple testing using the false discovery rate procedure [17], 30 (27%) out of 112 polymorphic AFLP markers that were detected in the admixed population deviated from a model of neutral introgression (Figure 3). Of these, 19 and 11 markers showed steeper and shallower genomic clines than expected given neutral introgression, respectively (see Additional File 2). 

### Morphological analyses

The first and second axis of principal component analysis explained 40.2 and 22.4% of the variation, respectively, and the measured flower characters allowed distinguishing unambiguously the two parental taxa from each other and *O. militaris* individuals from plants of the hybrid zone, but failed to separate pure *O. purpurea* individuals from putative hybrids (see Additional File 3).

The number of flowers was significantly different (*F*_2,124_ = 22.58, *P* < 0.001) between pure *O. purpurea* (35.8 ± 1.6), *O. militaris* (19.7 ± 1.4) and putative hybrids (26.5 ± 1.2). All other flower traits were also significantly different between pure *O. militaris*, *O. purpurea* and the putative hybrids (Table 2). In four out of eleven traits (length of the outer perianth segment, width of the torso, width of the leg and width of the labellum), plants from the hybrid zone were intermediate between the two parental taxa. In three out of eleven traits (spur width, length of the arm and length of the leg) plants in the hybrid zone were morphologically more extreme than either parent (Table 2). In three flower traits (spur length, width of the outer perianth segment, and length of the torso) plants from the hybrid zone resembled *O. militaris* more than *O. purpurea* (Table 2). Only in the width of the arm and the width of the labellum hybrids resembled *O. purpurea* more than *O. militaris*.

**Table 2:** **Means and standard errors of phenotypic traits related to flower morphology in pure *O. militaris, *pure *O. purpurea *and the hybrid zone**

**Flower trait**	***Orchis militaris***	**Hybrid zone**	***O. purpurea***
Spur length	4.97 ± 0.09^a^	5.28 ± 0.09^a^	5.92 ± 0.16^b^
Spur width	1.87 ± 0.03^b^	1.66 ± 0.03^a^	2.38 ± 0.07^c^
Length outer perianth segment	12.97 ± 0.13^a^	10.66 ± 0.13^b^	8.97 ± 0.23^c^
Width outer perianth segment	4.62 ± 0.07^ab^	4.77 ± 0.07^b^	4.30 ± 0.12^a^
Length torso	10.05 ± 0.12^a^	9.58 ± 0.12^a^	8.62 ± 0.17^b^
Width torso	1.75 ± 0.08^a^	2.90 ± 0.08^b^	4.05 ± 0.15^c^
Length arm	8.05 ± 0.18^a^	10.10 ± 0.18^b^	8.81 ± 0.22^a^
Width arm	1.07 ± 0.05^a^	1.83 ± 0.05^b^	1.99 ± 0.08^b^
Length leg	10.94 ± 0.20^a^	13.36 ± 0.20^b^	11.39 ± 0.20^a^
Width leg	2.98 ± 0.13^a^	4.49 ± 0.13^b^	5.46 ± 0.17^c^
Width labellum	8.07 ± 0.24^a^	11.14 ± 0.24^b^	12.14 ± 0.33^b^

All values are given in mm. Values with different letters in superscripts are significantly different at the α = 0.05 level.

**Figure 4:**
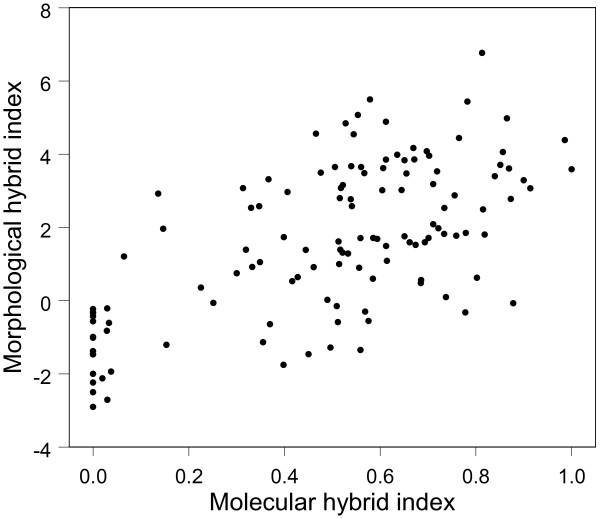
**Correlation between molecular and morphological hybrid indices in a hybrid population of *Orchis militaris *and *O. purpurea. ***

The canonical discrimant analysis yielded the function (eigenvalue = 12.46, χ^2^ = 146.86, canonical correlation = 0.96, *P* < 0.001): *D* = −0.610C + 0.521 G + 1.180 J. Based on this function, individuals of *O. militaris* received negative values, while those of *O. purpurea* had positive values (*O. militaris* = −3.47, *O. purpurea* = 3.47). Morphological hybrid indices calculated for plants of the hybrid population corresponded strongly with the molecular hybrid indices (*r* = 0.65, *P* < 0.001; Figure 4).

### Relationship between fitness and phenotypic traits

**Figure 5:**
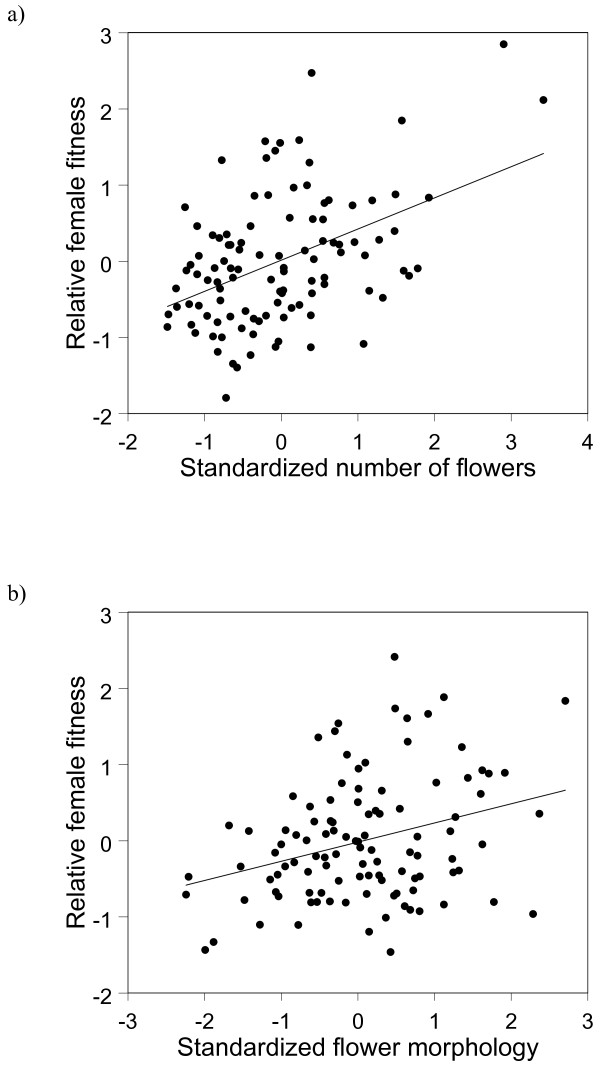
**Standardized linear phenotypic selection gradient for a) the number of flowers and b) floral morphology in a hybrid population of *Orchis militaris *and *O. purpurea *.** The selection gradient is illustrated with an added-variable plot, in which the residuals from a linear regression model of relative fitness on all traits except the focal trait are plotted against the residuals from a regression model of the focal trait on the other traits.

In the hybrid population, the number of fruits produced by a single plant varied between 0 and 22 fruits (mean: 5.3), corresponding to percentages fruit set of 0 and 61.9% (average 18.3%). The proportion of viable seeds varied between 0 and 0.89 (average: 0.26). Significant selection on the number of flowers (*β* = 0.411 ± 0.081, *t* = 5.080, *P* < 0.001) and the first PCA axis (*β* = 0.252 ± 0.076, *t* = 3.319, *P* = 0.001) were found (Figure 5). The variance inflation factors were small (< 1.2), indicating that there were no problems with collinearity.

## Discussion

### Hybridisation between *O. militaris* and *O. purpurea*

Species integrity between closely related sympatric species strongly depends on the strength of reproductive isolation. If parental species are not sufficiently reproductively isolated, the survival of pure parentals may become a challenge when occurring in close proximity. Using detailed morphological and molecular data, we showed that hybridization and genetic admixture had occurred in a population where *O. purpurea* and *O. militaris* grew in sympatry. Our data further showed that hybridization had occurred beyond the F_1_ generation, confirming previous reports that have shown that hybrid swarms can arise between these two species [8]. Across the entire genus *Orchis*, the formation of hybrid swarms is rare, and has been reported in only three other species combinations (*O. militaris* – *O. simiaO. mascula* – *O. pauciflora* and *O. anatolica* – *O. quadripunctata*) [8]. Nonetheless, some pure individuals were still present in the studied population, indicating that the parental species are sufficiently isolated to survive the challenge of sympatry.

In species that display overlap in phenology, reproductive isolation can be achieved by specific pollinators, post-pollination barriers or a combination of both. Because *O. purpurea* and *O. militaris* are pollinated by the same suite of generalist pollinators (mostly bees, bumblebees, and flies) [18,19] and display considerable overlap in flowering time, frequent exchange of pollen between the parental species is likely. Moreover, experimental pollinations between *O. purpurea* and *O. militaris* also showed that both species are capable of producing high levels (> 90%) of fruit set when they are pollinated with pollen of the other species [15]. These findings also comply with the fact that they have the same chromosome number (2*n* = 42) [8,20], allowing them to cross relatively easily. Therefore, the occurrence of pure *O. purpurea* and *O. militaris* individuals is more likely the result of late-acting post-mating barriers that limit crossbreeding between the hybrid and parental types or that lead to hybrid sterility.

The evolution of hybrid sterility has been shown to have played an instrumental role in determining reproductive isolation in food-deceptive orchids, as only two out of seven hybrid combinations were able to produce viable seeds when hybrids were backcrossed with parental plants [7]. In the studied population, average fruit set was similar to that observed in allopatric populations of *O. purpurea*[21], indicating that barriers to cross-breeding between hybrid plants and parental types were weak. On the other hand, fruits contained only about 25% of viable seeds, which is significantly lower than that observed in pure individuals, suggesting that the lower fitness of hybrids may to some extent have contributed to the maintenance of species integrity. However, since seed germination and protocorm development in orchids are dependent on suitable mycorrhizal fungi, and because both species and their hybrids associated with specific sets of mycorrhizal fungi [15], the patchy distribution of mycorrhizal fungi that are able to support germination of both pure and hybrid seeds may have further contributed to the maintenance of pure parentals. Seed germination experiments have indeed shown that germination of pure *O. militaris* seeds was mostly restricted to areas where pure *O. militaris* plants were growing, whereas hybrid seeds and seeds from pure *O. purpurea* mainly germinated in areas where *O. purpurea* was most abundant [16].

### Asymmetric introgression

Bayesian admixture analyses using STRUCTURE indicated that introgression was asymmetric with *O. purpurea* contributing more to the genomic composition of the hybrids than *O. militaris*. Hybrid indices also covered the entire range between 0 and 1, but there was an abrupt change between 0.1 and 0.3, suggesting that backcrosses with *O. militaris* were rare. Analyses using NEWHYBRIDS indeed showed that only very few backcrosses with *O. militaris* were present in the population. These results were corroborated by the morphometric analyses, which also failed to unambiguously distinguish hybrids from pure *O. purpurea* plants based on flower characteristics. However, failure to distinguish hybrid plants from pure *O. purpurea* plants may also be explained by the fact that flowers of *O. purpurea* display considerable variation and that therefore clustering of individuals based on morphological data was less stringent than that based on molecular data. Nonetheless, our data on individual characters of the parental species were remarkably similar to those presented for different allopatric populations of *O. purpurea* and *O. militaris* in the UK [22]. For example, the average width of the leg, which can be considered as taxonomically useful character clearly distinguishing the hybrids from the parental species [22], varied between 3.18 and 3.48, and between 4.28 and 5.35 in British populations of *O. militaris* and *O. purpurea*, respectively, whereas it was 2.98 and 5.46 in this study. Therefore, failure to distinguish pure individuals from hybrids most likely reflects severe mixing rather than the use of uninformative characters.

Asymmetric introgression can arise when the two parental species show different abundances or when parental species and hybrids display spatial segregation in the population, so that first-order hybrids will mate more frequently with the most abundant parent, or with the parental species it grows closest next to. In our previous analysis, we have shown that the spatial distribution of *O. militaris* was not related to that of hybrids or pure *O. purpurea* individuals, but that hybrids and pure *O. purpurea* individuals occupied the same area. Hybrids and pure *O. purpurea* individuals also showed similar mycorrhizal association patterns, whereas the community of mycorrhizal fungi associating with pure *O. militaris* plants was significantly different. Moreover, *O. militaris* plants showed a highly clustered distribution pattern [16], which may have favored pure, intraspecific pollination. In contrast, the distribution of F1 hybrid plants largely overlapped with that of backcrosses to *O. purpurea* and pure *O. purpurea* individuals, potentially contributing to pollen flow between hybrids and pure *O. purpurea* plants.

Our results further indicated that in the absence of other forms of selection, plants with large floral displays and thus resembling more *O. purpurea* and/or plants with floral traits similar to those of flowers of *O. purpurea* were favored. The large number of backcrosses with *O. purpurea* is in line with this observation. These results thus suggest that floral characters of *O. purpurea* are selectively favored in the hybrid zone. Genomic clines analysis also showed that about 27% of all AFLP markers deviated from neutral expectations, suggesting that they may introgress less (or more) frequently than expected under neutrality [23]. Such deviations can be interpreted as evidence of selection and suggest that gene movement across the hybrid zone is not fully random. Depending on the direction, deviations can either represent an increase in introgression rates (likely affecting loci that are advantageous to the population) or a decrease in introgression rates (likely affecting loci that are ecologically disadvantageous or that act epistatically). We have shown that about 60% of all markers that deviated from neutrality showed evidence for an increase in introgression rates.

## Conclusions

The analyses of morphometric and molecular data provide evidence for substantial introgression between *Orchis militaris* and *O. purpurea*. They also indicated that this hybrid population was dominated by individuals resembling more *O. purpurea*. Moreover, selection analyses based on cumulative female reproductive success showed that *O. purpurea*-like individuals were selectively favored. Nonetheless, a substantial amount of pure *O. militaris* individuals was observed. Probably, the highly clustered spatial distribution patterns and spatially restricted seed germination, most likely mediated by patchy distributions of suitable mycorrhizal fungi, contributed to maintenance of genetic integrity in this species. Future research should therefore incorporate associations with mycorrhizal fungi to understand speciation in orchids.

## Methods

### Study species

*Orchis militaris* and *O. purpurea* are two orchid species that belong to the anthropomorphic group of species within the genus *Orchis*[24] or to the subgenus *Orchis*[8]. *O. purpurea* has a predominantly Mediterranean distribution [25], although some isolated populations can be found in the UK, Belgium and The Netherlands, where the species reaches the northern border of its distribution range. *O. militaris*, on the other hand, shows a more continental distribution, occurring from the Atlantic Coast as far east as Mongolia [18]. The two species have slightly different habitat preferences. Whereas *O. purpurea* is essentially a scrub or woodland plant that is mainly found in deciduous woodland (rarely in coniferous woodland), dense chalk scrub or coppice [25], *O. militaris* occurs more frequently in grazed dry meadows or in calcareous pastures [8]. However, in calcareous grassland immediately bordering forests or grasslands with little scrub encroachment, both species are often found growing together.

*O. purpurea* and *O. militaris* are both food deceptive species and pollinated by generalist pollinators, most often bumblebees or bees, but small flies and butterflies have also been reported visiting the flowers of both species [15,18]. The species display considerable overlap in flowering time: whereas flowering in *O. purpurea* starts at the beginning of May and lasts until the beginning of June [25], *O. militaris* usually starts flowering two weeks later (mid-May) and flowering lasts until mid June [18]. Flowers are conspicuously purple and show little variation in *O. militaris*, whereas in *O. purpurea* the flowers can display considerable variation, particularly in the shape and colour of the labellum and the helmet. The commonest variant has a dark reddish-violet or brownish-purple helmet and a pale-coloured labellum [26], but the helmet can also be green flecked with purple, dull rose outside and green within and with pale rose markings, greenish-white with pink veins, or reddish purple. The colour of the labellum can vary from pure white with extremely faint spots to intense pink with bright purple spots [8].

### Study site and sampling

The study was conducted in a calcareous grassland located in a local nature reserve near Eben-Emael (Belgium) (50°46’N 5°40’E, 210 m a.s.l.). This site had been severely overgrown by shrubs and trees, but was recently restored by cutting down all woody vegetation. As a result, individuals of *O. militaris* and *O. purpurea* grow in close proximity, which resulted in hybridization at the study site [16]. In May 2011, a total of 140 plants was sampled in a 20 x 30 m plot (see ref [16] for more details). For each of the 140 sampled plants, we also determined the fruit set and the seed vaibility. In July, when fruits were mature, the number of fruits per plant was counted. Fruit set was determined as the number of fruits divided by the number of flowers per plant multiplied by 100. From each plant that produced fruits, three fruits were harvested (or all fruits if less than three were produced) and for each fruit, the proportion of viable seeds was determined. To distinguish viable from non-viable seeds, a batch of *c*. 200 seeds per fruit was colored with tetrazolium using a modified staining technique [27].

Additionally, leaf samples were collected from 30 individuals in pure populations of both species. One population (*O. purpurea*) was located in a deciduous forest in Voeren about 15 km from the study plot, whereas samples of *O. militaris* were taken from a large nearby population growing in species-rich calcareous grassland in The Netherlands (distance to the hybrid population: 16 km). In these populations, the presence of the other orchid species has never been recorded, and therefore these plants were used as reference taxa in genome-wide admixture analyses [28]. Young leaf material was collected and immediately frozen in liquid nitrogen for AFLP analysis. For each plant, the number of flowers was counted and two or three flowers were harvested at the peak of flowering, stored in a denatured ethanol preservative (70%) and transported to the laboratory for morphometric analyses.

### DNA extraction and AFLP analysis

DNA extraction and AFLP analysis followed the same protocol as described in ref [16]. Briefly, leaf material was freeze-dried for 48 h and homogenized with a mill (Retsch MM 200) to fine powder. Total DNA was extracted from 30 mg of freeze-dried leaf material using the Nucleospin® 96 Plant Kit (Machery-Nagel). DNA concentrations were estimated using a NanoDrop ND-1000 spectrophotometer running software v3.0.1 (NanoDrop Technologies) following the manufacturer’s instructions.

AFLP analysis was carried out according to [29], using commercial kits and following the protocol of [30]. The enzymes *Eco*RI and *Mse*I were used for DNA digestion. Each individual plant was fingerprinted with three primer combinations: *Eco*RI-ACC/*Mse*I-CCTA, *Eco*RI-AGG/*Mse*I-CTGG and *Eco*RI-AGG/*Mse*I-CTAG. Fragment separation and detection took place on an ABI Prism 3130xl capillary sequencer. GeneScan 500 ROX-labelled size standard (Applied Biosystems) was used for fragment sizing. The fluorescent AFLP patterns were scored using GeneMapper version 3.7 (Applied Biosystems). We scored the presence or absence of each marker in each individual plant. Monomorphic markers were excluded from all further analyses. Reproducibility was assessed as described previously [16].

### Morphological measurements

To assess differences in morphological trait expression between parental species and individuals from the hybrid zone, eleven flower characters were used. To this end, each flower was dissected and digital photographs were taken. Using the image analysis software imagej 1.33, the following measurements were recorded for each flower (see Additional File 4 for more details): A) spur length, B) spur width, C) length of the outer perianth segment, D) width of the outer perianth segment, E) length of the torso, F) width of the torso, G) length of the arm, H) width of the arm, I) length of the leg, J) width of the leg and K) width of the labellum.

### Data analysis

#### Genetic diversity and differentiation

Based on the AFLP data, genetic diversity of the pure *O. purpurea* and *O. militaris* populations and the hybrid population were calculated using AFLPsurv version 1.0 [31]. Estimates of allelic frequencies at AFLP loci were calculated using the Bayesian approach with a non-uniform prior distribution of allele frequencies [32]. After estimating allele frequencies, the percentage of polymorphic loci (*P*%) and unbiased estimates of genetic diversity (*H*_j_) were calculated following [33]. To estimate genetic differentiation, the overall *F*_ST_ value and pairwise *F*_ST_ values were calculated after [33]. 99% confidence intervals were determined by random permutation (*n* = 5000) of individuals among populations. Genetic structure was assessed using Principal Coordinate Analysis (PCoA) calculated in GenAlEx[34] and the Bayesian clustering analysis implemented in Structure[35,36]. Structure uses a model-based clustering method to assign individuals to groups in which deviations from Hardy–Weinberg equilibrium and linkage equilibrium is minimized. Individuals assigned to two sources with non-trivial probabilities were considered putative hybrids. Structure was run ten times at *K* = 1–7 assuming no prior population information, with correlated allele frequencies and admixture, 200 000 burn-in cycles and 1 000 000 MCMC. The value of *K* that best fits our data was selected using the Δ*K* statistic [37].

#### Genome-wide admixture and calculation of hybrid index

To assess genome-wide admixture we used a simple hybrid index that is estimated based on information from dominant molecular markers [28]. This method was preferred to Bayesian admixture analyses because, unlike Bayesian admixture proportions, it uses the parental populations to estimate parental allele frequencies and because interpretation of the results is straightforward when parental taxa are well-defined [28]. The AFLP data obtained from the pure populations were used as parental data, whereas the AFLP data from the hybrid zone were entered as putatively admixed individuals. Assessment of genome wide admixture was done using the est.h function incorporated in the R program Introgress[38]. This function renders for each potentially admixed individual a maximum likelihood hybrid index estimate with its 95% confidence interval [38]. Hybrid indices vary between zero and one, corresponding to pure individuals of the alternative species and reference species, respectively. In addition, the program NEWHYBRIDS [39] was used to estimate nuclear admixture proportions and patterns of introgression. The model implemented in NEWHYBRIDS assumes that the samples analyzed are composed by two pure parental species and hybrids. Under this model, *q* describes posterior probabilities for each individual, which are classified as parental purebreds, F1, F2 and backcross categories. As in the admixture analysis outlined above, individuals from the pure populations were incorporated in the analysis. Each analysis was run independently for three times, starting with a different random number of seeds and for 10^5^ iterations of MCMC chains after 10^4^ burn-in steps.

In order to understand whether introgression for single markers deviated from neutral expectations, we estimated genomic clines for each locus in the admixed population and using the pure populations as reference populations [40]. We used the parametric procedure in the software program Introgress to perform significance testing for departures from neutral expectations for genomic clines [38]. To correct for multiple independent tests, we used the false discovery rate procedure [17].

#### Morphometric analyses

A Principal Component Analysis (PCA) was conducted to describe the overall differences in flower morphology among individuals from pure populations and individuals from the hybrid zone. Because the first two axes were found to be representative of the higher order axes, only PCA scores of the first two axes were plotted. A stepwise canonical discriminant analysis was used to describe the average floral morphology of each plant in the hybrid population and to obtain an index of morphological variation among plants [41]. Trait measurements of the pure populations were used to derive the discriminant function [10]. A stepwise procedure was used to find the linear combination that best characterizes the two species. An *F*-value of 3.84 was used to enter a variable and an *F*-value of 2.71 to remove it. The resulting indices of morphological variation were related to molecular hybrid indices using a Spearman rank correlation.

A one-way Analysis of Variance (ANOVA) was used to investigate whether morphological traits were significantly different between individuals from the pure populations and putative hybrids. For the sympatric population, only individuals with a hybrid index ranging from 0.1 and 0.9 were used. The same test was used to investigate whether the number of flowers per flowering stalk differed between pure and putative hybrids. Tukey’s post hoc test was used to determine which traits were significantly different between the three groups.

#### Estimating selection

Selection was estimated following [42], using multiple regression analyses with relative fitness (individual fitness divided by mean fitness) as the response variable and standardized trait values (with a mean of 0 and a variance of 1) as explanatory variables. To reduce the number of floral traits and to avoid problems with collinearity, scores of the first two axes of a principal component analysis involving all measured floral traits were used. A cumulative fitness measure (the number of fruits*seed viability) was used as dependent variable in the regression analyses. We also incorporated plant size (measured as the number of flowers) in the analysis. Selection gradients were estimated by calculating partial regression coefficients from the multiple regression. Finally, added-variable plots, in which the residuals from a linear regression model of relative fitness were on all traits except the focal trait are plotted against the residuals from a regression model of the focal trait on the other traits, were created. All analyses were conducted using SPSS version 16.0.

## Competing interests

The authors declare that they have no competing interests.

## Authors’ contributions

HJ, RB and OH conceived the study, HJ and RB carried out the field work, RB performed the morphometric analyses and HJ performed the data analyses. IRR coordinated the molecular genetic studies. All authors contributed to the writing of the manuscript and they all read and approved the final manuscript.

## Supplementary Material

Additional file 1 **STRUCTURE analysis of 113 AFLP markers scored in 200 individuals originating from a pure *Orchis militaris *populations, pure *O. purpurea *population and a hybrid population using different values of *K. ***Additional File 2 Figure S2a gives the most likely number of clusters. Additional File 2: Figure S2b gives the results for *K* = 3. Click here for file

Additional file 2 **Figure S2.** Genomic clines for introgression of AFLP markers from a hybrid zone between * Orchis militaris * and * Orchis purpurea. * The name of each locus is given and the * P *-value for the test of departure from neutrality. The solid line is the estimated cline based on individuals that lacked the dominant AFLP marker. Click here for file

Additional file 3 **Principal component plot of the first and second axis for morphometric data from putative hybrids and both parents (*O. militaris *and *O. purpurea *).** Click here for file

Additional file 4 **Floral traits measured in 200 individuals of *O. militaris, O. purpurea, *and their putative hybrids. Letters refer to traits listed in Materials and Methods.** Click here for file
