# Supplementary material for: Asymmetric gene introgression in two closely related Orchis species: evidence from morphometric and genetic analyses
Source: BMC Evol Biol. 2012 Sep 12;12:178. doi: 10.1186/1471-2148-12-178 (PMC3523012; doi:10.1186/1471-2148-12-178)

Fig. S1 STRUCTURE analysis of 113 AFLP markers scored in 200 individuals originating from a pure *Orchis militaris* populations, pure *O. purpurea* population and a hybrid population using different values of *K*. Fig. S1a gives the most likely number of clusters. Fig. S1b gives the results for *K* = 3.

a)

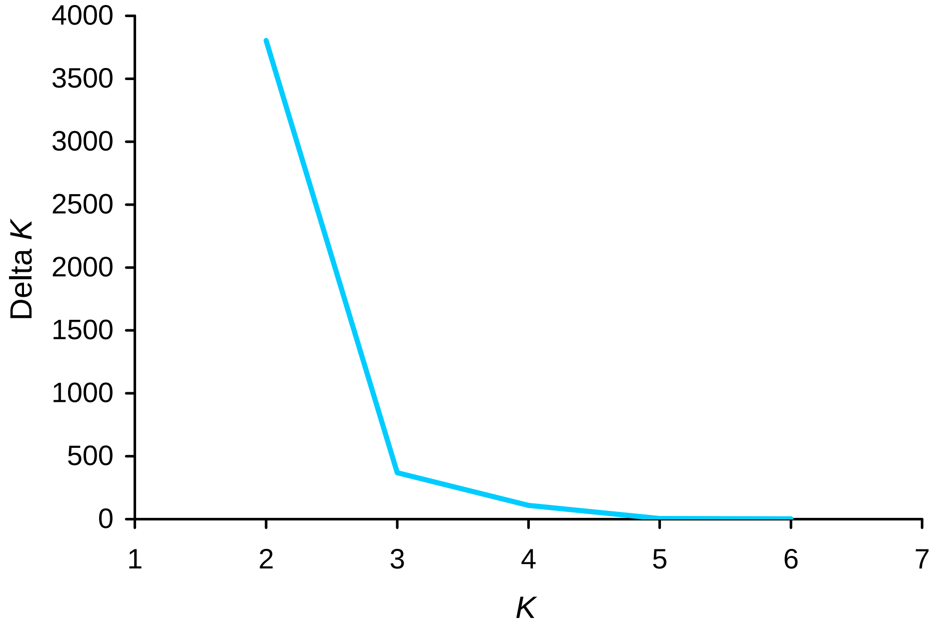

b)

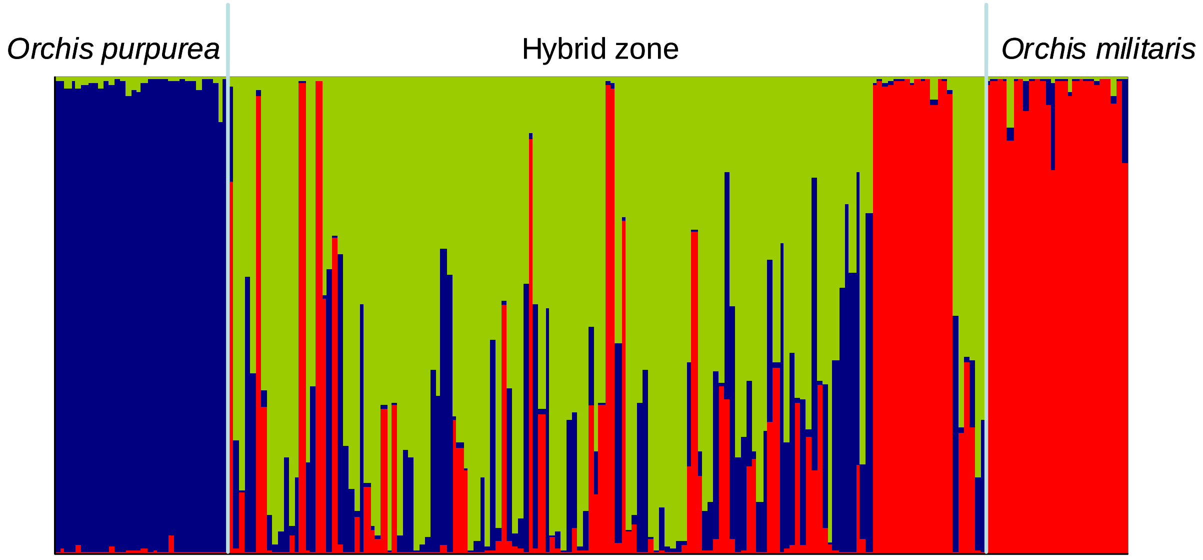

Supplement: Additional file 1 — STRUCTURE analysis of 113 AFLP markers scored in 200 individuals originating from a pure Orchis militaris populations, pure O. purpurea population and a hybrid population using different values of K. Additional File 2 Figure S2a gives the most likely number of clusters. Additional File 2: Figure S2b gives the results for K = 3. [file 1471-2148-12-178-S1.pdf]
