# Supplementary material for: Asymmetric gene introgression in two closely related Orchis species: evidence from morphometric and genetic analyses
Source: BMC Evol Biol. 2012 Sep 12;12:178. doi: 10.1186/1471-2148-12-178 (PMC3523012; doi:10.1186/1471-2148-12-178)
Supplement: Additional file 2 — Figure S2. Genomic clines for introgression of AFLP markers from a hybrid zone between Orchis militaris and Orchis purpurea. The name of each locus is given and the P -value for the test of departure from neutrality. The solid line is the estimated cline based on individuals that lacked the dominant AFLP marker. [file 1471-2148-12-178-S2.pdf]

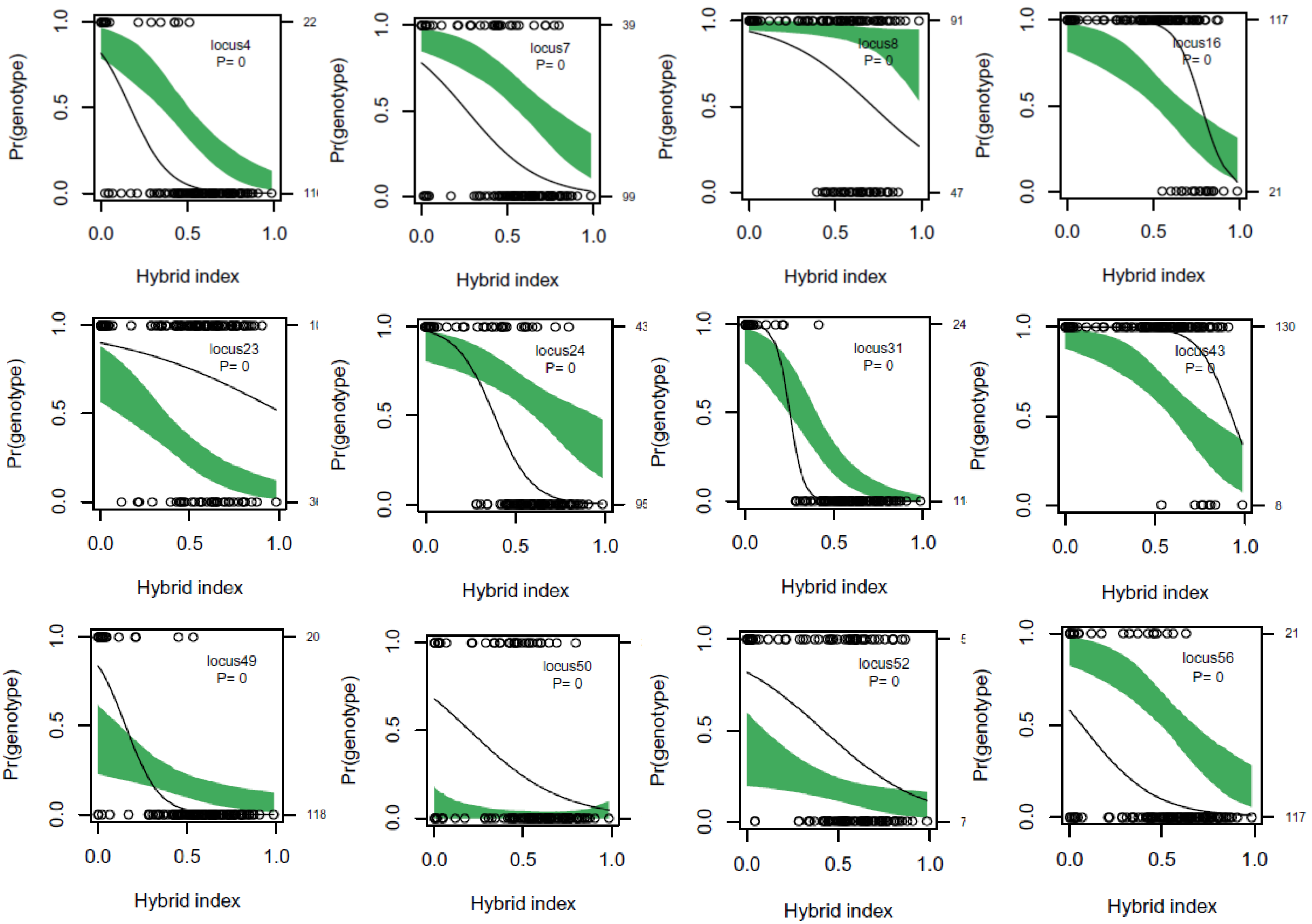

locus4
P= 0
locus7
P= 0
locus8
P= 0
locus16
P= 0
locus23
P= 0
locus24
P= 0
locus31
P= 0
locus43
P= 0
locus49
P= 0
locus50
P= 0
locus52
P= 0
locus56
P= 0
Pr(genotype)
Hybrid index

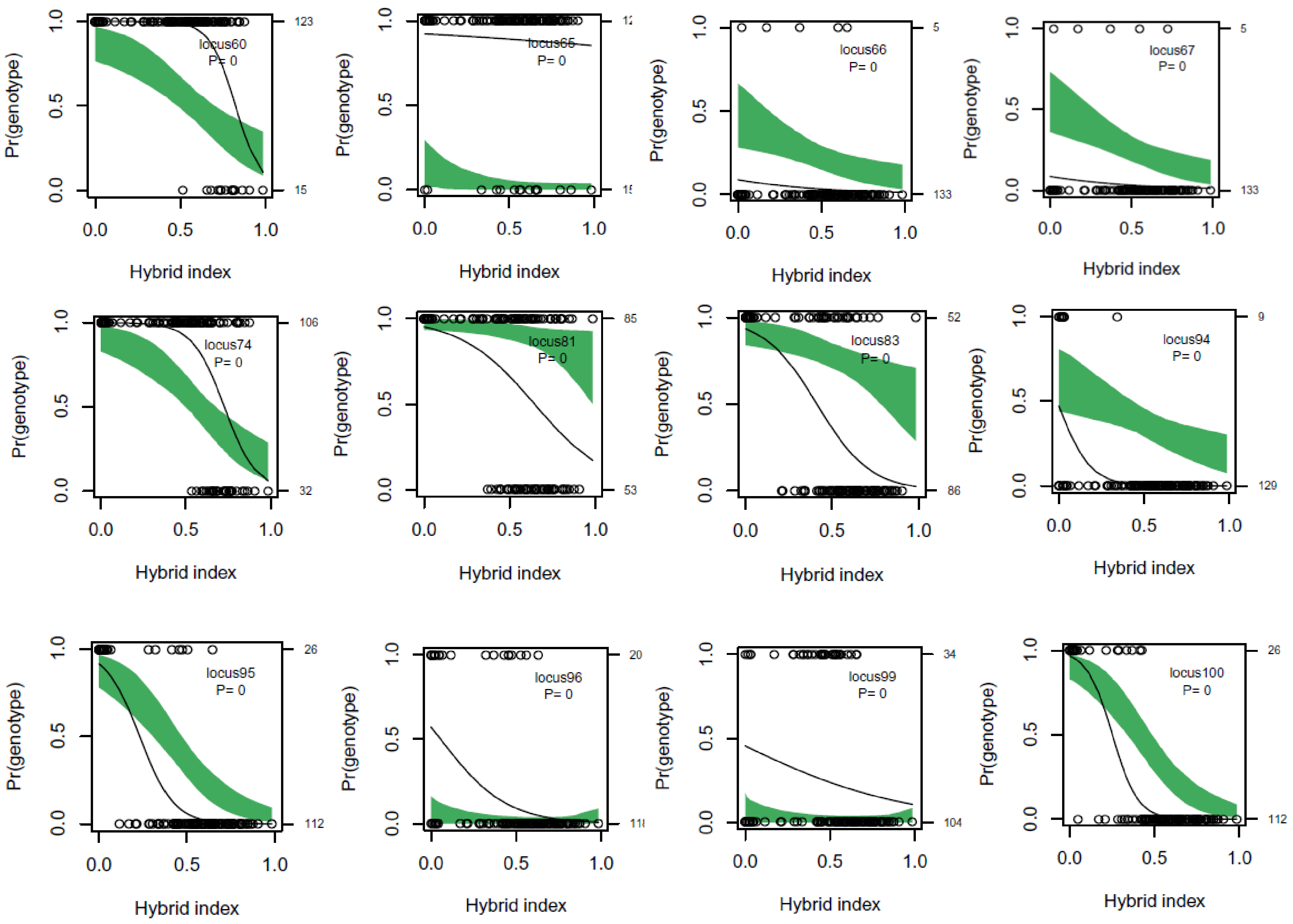

Pr(genotype)
Hybrid index
0.0
0.5
1.0
locus60
P= 0
123
15
locus65
P= 0
locus66
P= 0
5
133
locus67
P= 0
5
133
locus74
P= 0
106
32
locus81
P= 0
85
53
locus83
P= 0
52
86
locus94
P= 0
9
129
locus95
P= 0
26
112
locus96
P= 0
20
locus99
P= 0
34
104
locus100
P= 0
26
112

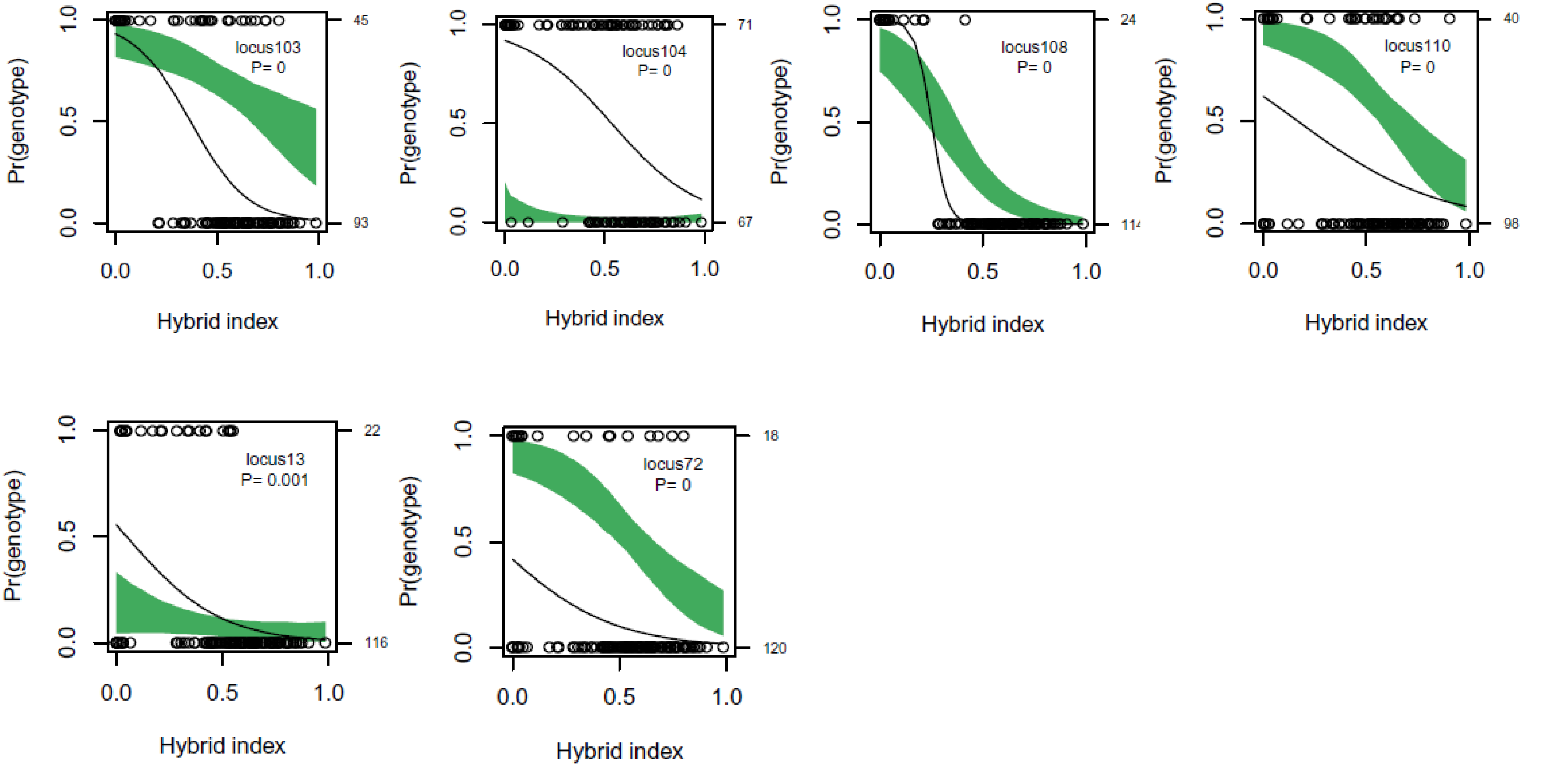

Additional File 2 Genomic clines for introgression of AFLP markers from a hybrid zone between *Orchis militaris* and *Orchis purpurea*. The name of each locus is given and the *P*-value for the test of departure from neutrality. The solid line is the estimated cline based on individuals that lacked the dominant AFLP marker.
