# Supplementary material for: Asymmetric gene introgression in two closely related Orchis species: evidence from morphometric and genetic analyses
Source: BMC Evol Biol. 2012 Sep 12;12:178. doi: 10.1186/1471-2148-12-178 (PMC3523012; doi:10.1186/1471-2148-12-178)

Additional File 3 Principal component plot of the first and second axis for morphometric data from putative hybrids and both parents (*O. militaris* and *O. purpurea*)

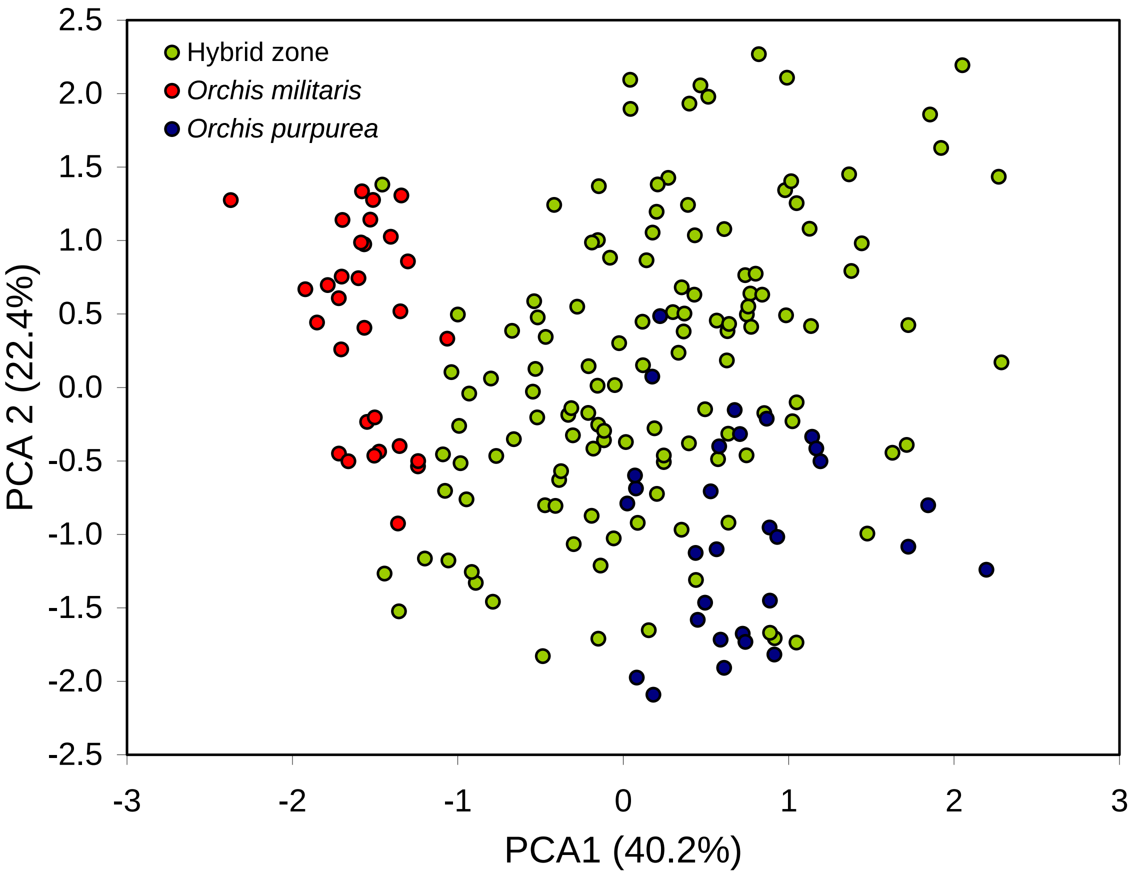

Supplement: Additional file 3 — Principal component plot of the first and second axis for morphometric data from putative hybrids and both parents (O. militaris and O. purpurea ). [file 1471-2148-12-178-S3.pdf]
