# Supplementary material for: Asymmetric gene introgression in two closely related Orchis species: evidence from morphometric and genetic analyses
Source: BMC Evol Biol. 2012 Sep 12;12:178. doi: 10.1186/1471-2148-12-178 (PMC3523012; doi:10.1186/1471-2148-12-178)

Additional File 4 Floral traits measured in 200 individuals of *O. militaris*, *O. purpurea*, and their putative hybrids. Letters refer to traits listed in Materials and Methods.

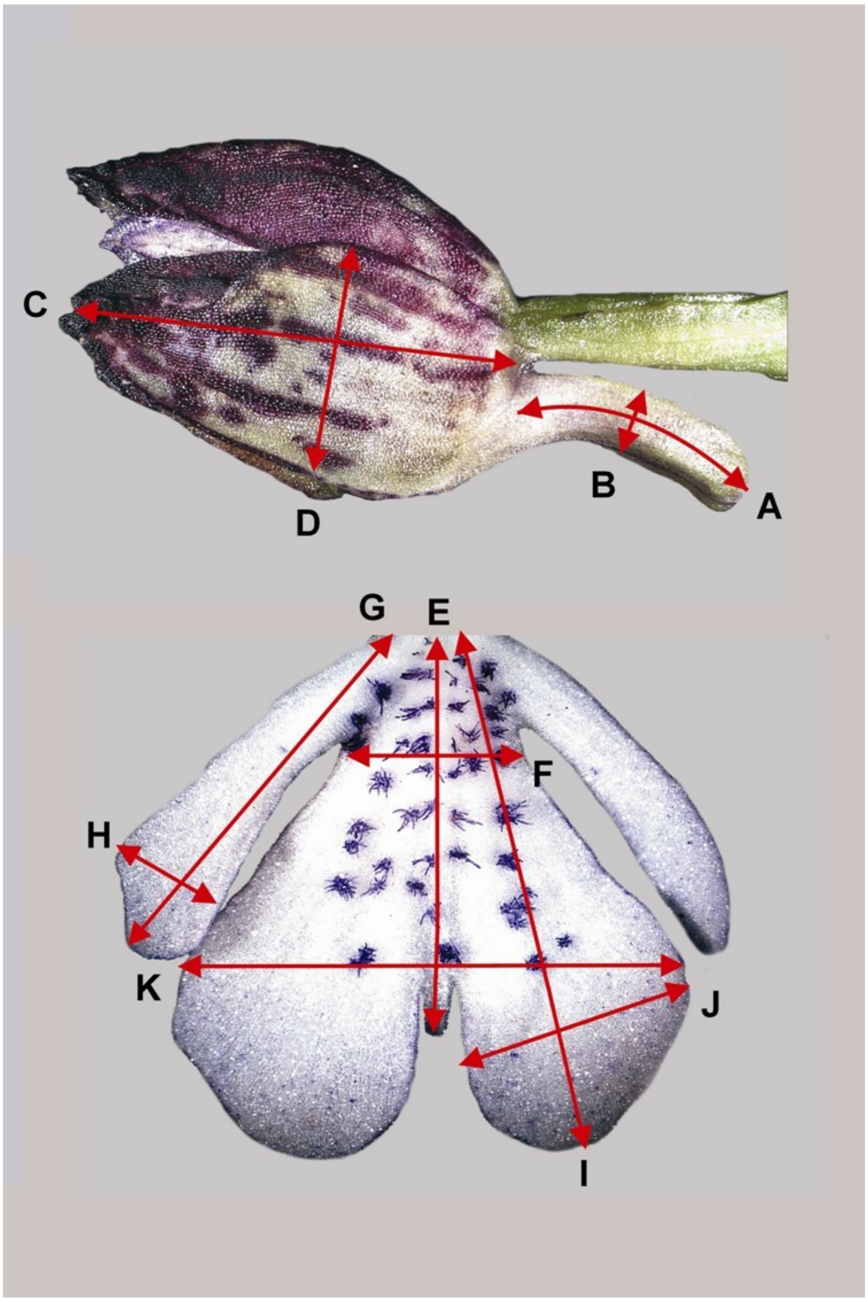

Supplement: Additional file 4 — Floral traits measured in 200 individuals of O. militaris, O. purpurea, and their putative hybrids. Letters refer to traits listed in Materials and Methods. [file 1471-2148-12-178-S4.pdf]
